# Phlegmonous gastritis complicated by abdominal compartment syndrome: a case report

**DOI:** 10.1186/s12893-020-00999-y

**Published:** 2021-01-04

**Authors:** Mana Modares, Mohammad Tabari

**Affiliations:** 1 Faculty of Medicine, University of Toronto, Toronto, ON Canada; 2 Department of Surgery, Scarborough Health Network, University of Toronto, 3030 Lawrence Avenue East, suite 414, Toronto, ON M1P 2T7 Canada

**Keywords:** Phlegmonous gastritis, Upper endoscopy, Abdominal compartment syndrome, Acute abdomen, Computed tomography

## Abstract

**Background:**

Phlegmonous gastritis (PG) is a rare, suppurative bacterial infection of the gastric wall, which may rapidly evolve into fatal septicemia. The etiology and pathogenesis are poorly understood; however, multiple risk factors have been cited in current literature. Most cases have been diagnosed at autopsy, and occasionally at laparotomy, as the clinical presentation is often variable.

**Case presentation:**

We report a case of a 67-year-old male presenting with intractable nausea, vomiting, and epigastric pain following an uneventful upper gastrointestinal (GI) endoscopy. Diagnostic workup including contrast tomography (CT) and endoscopic assessment was in keeping with PG. This was subsequently followed by development of abdominal compartment syndrome (ACS) and clinical deterioration necessitating surgical resection of the stomach.

**Conclusion:**

This case emphasizes the importance of early diagnosis of this potentially fatal infection that can follow endoscopic procedures and illustrates ACS and septic shock as serious complications. There is currently no consensus on the proper management of PG; however, in this case, a combination of surgery and antibiotics provided a favourable outcome. Limited number of cases of PG have been reported in literature, and to our knowledge, this is the first reported case of PG with subsequent ACS as an acute complication.

## Background

Phlegmonous gastritis (PG) is a rare disease characterized by suppurative bacterial infection of the gastric wall, which without prompt diagnosis and appropriate treatment, may rapidly evolve to a fatal systemic septicemia [1, 2]. PG affects the submucosa and muscularis propria layers of the stomach wall in a focal or diffuse manner [1]. It typically presents with upper gastrointestinal (GI) symptoms, such as nausea, vomiting, and hematemesis [1]. The exact etiology and pathophysiology of disease are poorly understood; however, multiple risk factors such as mucosal injury, alcoholism, achlorhydria, advanced age, prior gastric surgery or biopsy, and an immunocompromised state have been cited [1, 2]. Numerous bacterial organisms have been implicated as the most common pathogens; however, the *Streptococcus* genus accounts for approximately 67–75% of cases [1]. Diagnosis is difficult and requires a combination of clinical presentation, imaging, bacterial culture, and pathology [3].There is currently no consensus on the proper management of PG; however, mortality rates have been reported as high as 27% [1–3]. Therefore, early recognition and treatment with antibiotics is imperative.

To date, only six cases of post-endoscopy PG have been reported [4–9]. Patients presented with acute abdominal pain and/or nausea vomiting post-procedure, resulting in rapid deterioration [4–8]. Almost all patients and had to undergo total gastrectomy as their definitive management [4, 6–9]. We encountered such a case, which was complicated by septic shock and abdominal compartment syndrome (ACS). To our knowledge, this is also the first reported case of PG with ACS as an acute complication.

## Case presentation

**Fig. 1:**
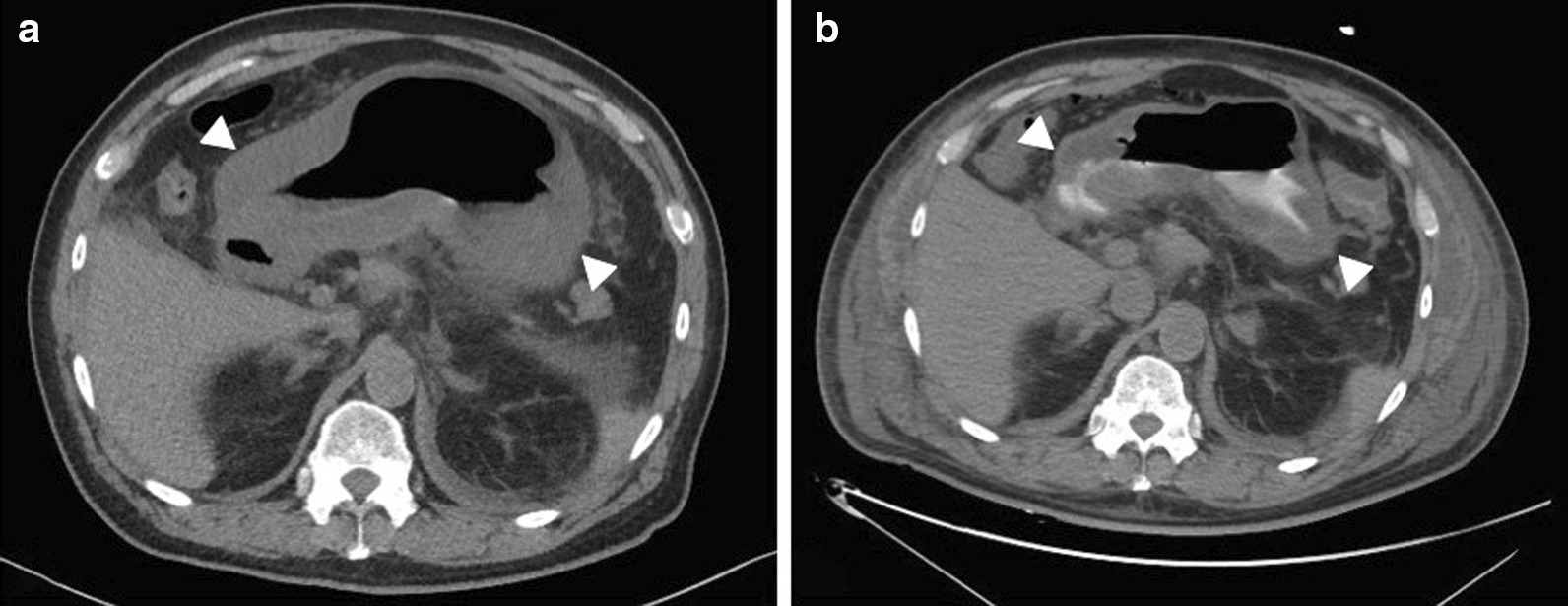
Computed tomography (CT). Axial view of the abdomen. **a** Typical findings of phlegmonous gastritis with diffuse gastric wall thickening (arrows), most pronounced distally. **b** Post-laparotomy demonstrates persistent diffuse gastric wall thickening (arrows)

A 67-year-old male with a history of type II diabetes mellitus and no previous surgical history underwent an uneventful outpatient upper GI endoscopy with a random biopsy as part of a diagnostic workup for anemia. Later that same evening, the patient developed intractable nausea, vomiting, and epigastric pain. The following day, he presented to a peripheral hospital with a white cell count (WBC) of 19,000/mm^3^ (reference range: 4500–11,000/mm^3^), temperature of 40.0 °C, and hypotension (95/65 mmHg). His condition rapidly deteriorated, which prompted transfer to the intensive care unit (ICU), where he was subsequently intubated the following day. The patient was resuscitated with 18 L of intravenous fluids and was placed on three classes of vasopressors. A computed tomography (CT) scan of the abdomen and pelvis did not demonstrate any evidence of hollow viscus perforation, but there was minimal ascites with a severe and diffusely thickened gastric wall (Fig. 1a). The patient was treated with antibiotics empirically. His blood culture grew group A streptococci; thus, he had developed bacteremia from the endoscopy the day before. He subsequently developed multiorgan failure with anuria and lactate of 6.5 mmol/L (reference range, 0.5–1 mmol/L) with arterial blood pH of 7.2, HCO_3_ of 12 mEq/L, and BE of − 12.5 (reference ranges, pH: 7.35–7.45, HCO_3_: 22–28 mEq/L, BE: − 5 to + 5 mEq/L). His abdomen was distended, and bladder pressure was 28 mmHg.

The patient was transferred to our centre for hemodialysis on the fourth day following the initial presentation. At this time, his bladder pressure had further risen to 36 mmHg (reference range, 0–5 mmHg). Ventilation was difficult, and his blood pressure remained at 60–80 mmHg systolic despite three vasopressors. He continued to demonstrate anuria with progressive abdominal distention and grimacing upon palpation. The patient was ASA class 5E and the decision was made for urgent life-saving laparotomy to decompress the abdomen with the provisional diagnosis of acute ACS.

In the operating room, there was evidence of diffuse gastric ischemia with areas of greyish-white and yellowish to dusky tan throughout the gastric wall. Moderate volume of cloudy ascites was present that was sent to cytology and numerous inflammatory cells were found. Abdominal fluid samples were sent for culture, which subsequently grew group A streptococci. The abdomen was left open with a vacuum-assisted closure (VAC) dressing. The patient was returned to the ICU to further stabilize prior to further surgical procedures. Hemodialysis was initiated the next day with intravenous immunoglobulins. Antibiotics and supportive care were continued in the ICU.

**Fig. 2:**
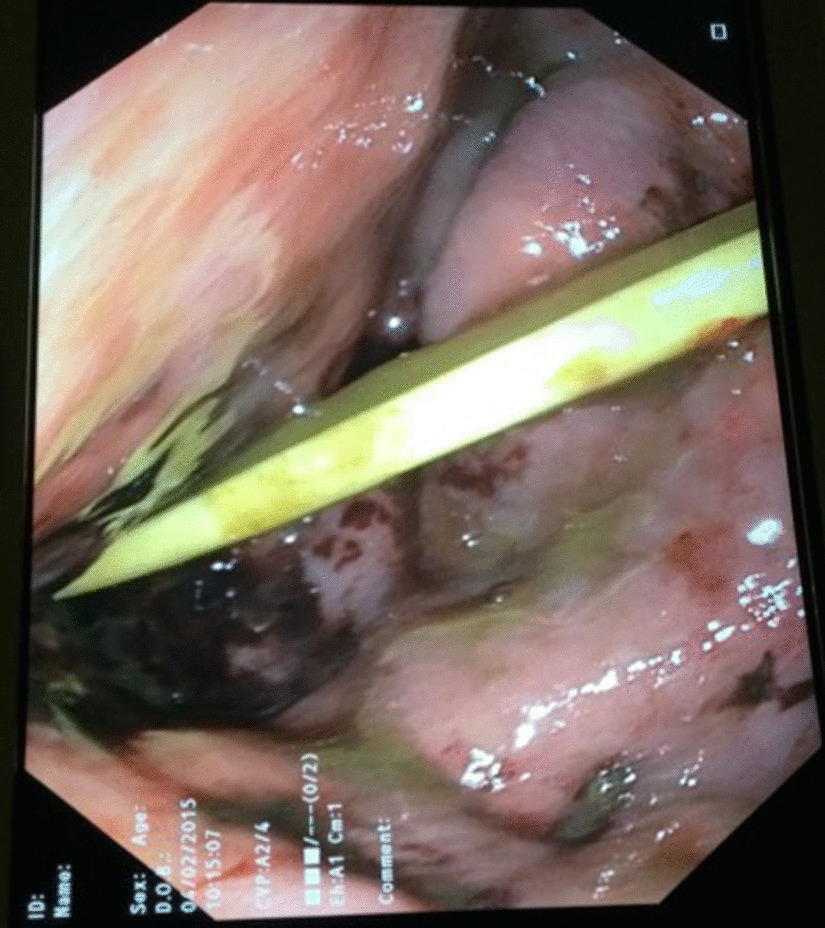
Endoscopic view of the stomach demonstrates ischemic mucosa with areas of ulceration, necrosis, and exudate

On the 6^th^ day after initial presentation he had stabilized further and was no longer on vasopressors. An upper GI endoscopy was performed, which demonstrated diffuse ischemic gastritis with areas of ulceration, necrosis, and exudate (Fig. 2). A CT scan of the abdomen and pelvis was repeated on the same day, again demonstrating diffuse and severe gastric wall thickening (Fig. 1b). The decision was made for total gastrectomy with a diagnosis of diffuse gastric ischemia with necrosis. Roux-en-Y esophagojejunostomy and jejunojejunostomy reconstruction was performed on the 7th day following presentation. A feeding jejunostomy tube was inserted. The abdomen was again left open 60% on VAC.

**Fig. 3:**
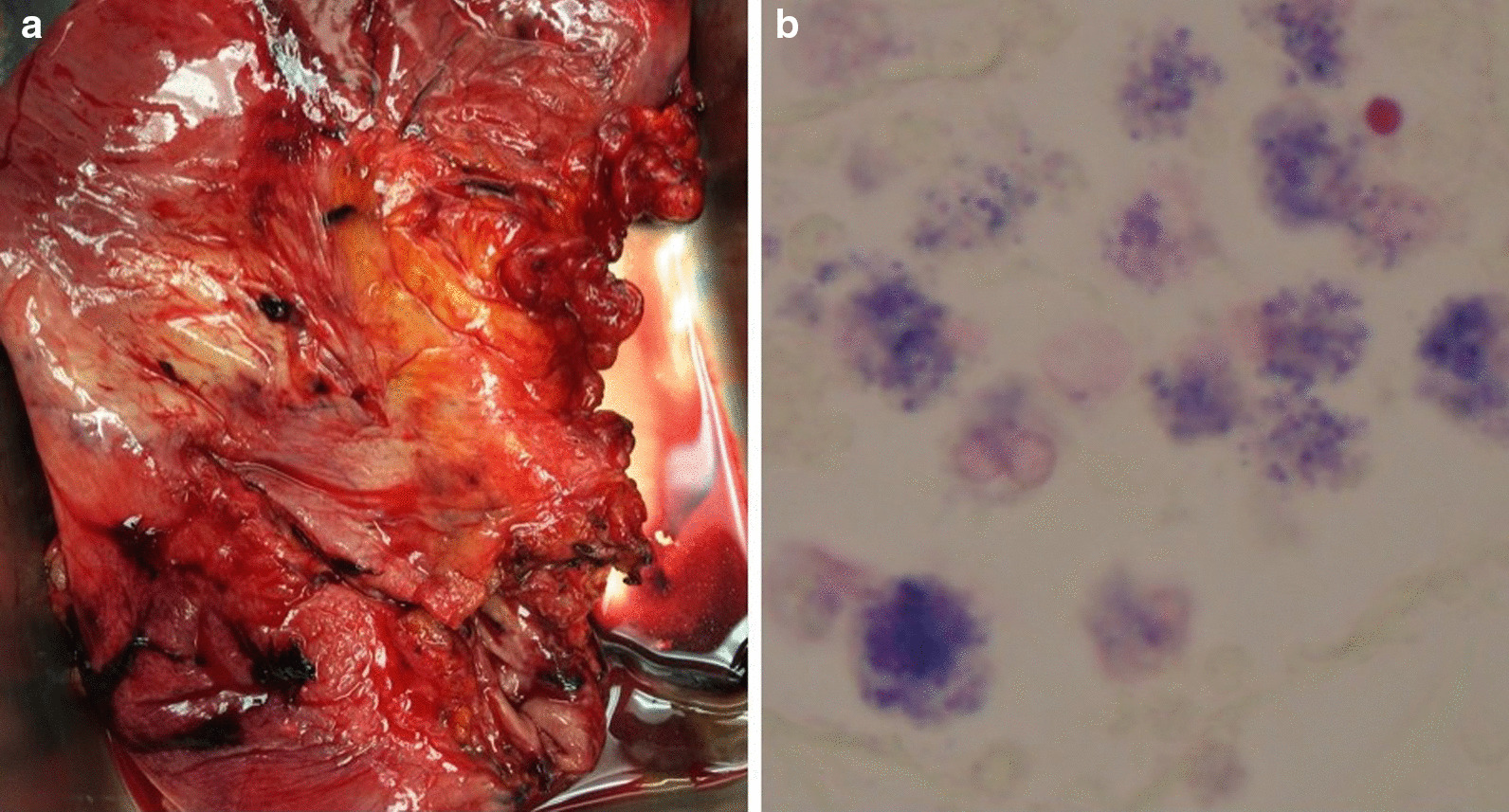
**a** Gross specimen. Gastric wall demonstrating areas of ischemia and hemorrhagic necrosis. **b** Gram stain photomicrograph of the gastric specimen showing intracellular positive (blue) granules consistent with gram positive microorganisms

The gross specimen demonstrated greyish-white to dusky tan appearance with patchy areas of necrosis and hemorrhagic mucosa (Fig. 3a). No definite focal lesion or perforation sites were identified. Pathology report demonstrated PG with diffuse transmural inflammation and extensive necrosis. Intracytoplasmic gram-positive granules were seen on gram stain (Fig. 3b). No *H. Pylori* was found.

On postoperative day (POD) 1, the patient’s condition markedly improved. Vasopressors were discontinued at the time. A CT scan on POD 3 did not demonstrate any anastomotic leakage. The patient continued to improve with normalization of WBC and lactate levels. On POD 9, he was extubated, and antibiotics were discontinued. Total parenteral nutrition and nasogastric feeding tubes were discontinued, and he was subsequently started on jejunal feeds. On POD 12, the patient was returned to the OR for closure of the abdominal wall as the abdomen was no longer edematous. There was a leak from jejunostomy tube on POD 20 and a stent was endoscopically inserted to resolve the issue. All other medications and interventions were tolerated well. He was on hemodialysis for 1.5 months until he was able to urinate. He had 3 months of recovery until he was well enough to go home. There were no issues at the follow-up visit.

## Discussion and conclusions

PG is a rare and often fatal entity characterized by suppurative bacterial infection of the gastric wall [1]. Our patient also had a history of type II diabetes mellitus, which is one of many predisposing factors [2]. A case of a foreign body injury causing PG in a patient with dementia was reported, highlighting the importance of considering cognitive impairment as a possible risk factor [10]. Furthermore, various routes of infection, including direct and hematogenous infection of the lumen, have been implicated and termed primary and secondary forms of PG, respectively [11, 12]. Lastly, there are localized and diffuse forms of the disease with the latter form demonstrating significantly higher mortality rates (10% vs. 54%) [13, 14]. Any mucosal injury can provide a route for bacterial penetration, and in this case, a background state of immunosuppression likely predisposed the patient to a fulminant bacterial infection [4]. Our case was classified as primary and diffuse.

Diagnostic investigations for PG include abdominal ultrasound, CT scan, endoscopy, or endoscopic ultrasound; upper endoscopy is considered the diagnostic gold standard [1, 2, 12]. Findings on endoscopy are typically thickened and edematous gastric mucosa with occasional purulent discharge, which is pathognomonic for PG [12]. The CT findings in this case showed severe diffuse thickening and edema of the gastric wall. Given poor renal function, no intravenous contrast was administered on either scan; however, the degree and distribution of wall thickening in this acute clinical setting is highly concerning for an advanced diffuse infectious and/or ischemic process. Histopathology demonstrated diffuse transmural inflammation, with extensive necrosis and intracytoplasmic gram-positive granules seen on the gram stain. Blood cultures grew group A streptococci, which is in agreement with the most common organism stated in the literature [1]. Finally, upper endoscopy revealed areas of tissue necrosis and exudate leading to the provisional diagnosis of PG. The diffuse type of PG has been characterized by dark red and diffuse gastric wall thickening, which can cause gastric cavity expansion and gastric wall perforation, thus requiring urgent intervention [14, 15].

In our patient, acute ACS developed as a result of the following risk factors: large fluid resuscitation, acidosis, presence of ascites, and sepsis, with subsequent multi-organ failure [16]. As seen in our patient, deterioration can be rapid if undiagnosed and untreated, leading to acute peritonitis and death [4]. Up to 50% of ICU patients are at risk of developing intra-abdominal hypertension (IAH) and 8% are at risk for ACS [17]. IAH is defined as intra-abdominal pressure (IAP) equal to or greater than 12 mmHg, and ACS is sustained IAP above 20 mmHg with new onset end-organ dysfunction [18]. Our patient had an IAP of 36 mmHg with clinical deterioration in the ICU leading to the diagnosis of ACS.

It has been reported that a prolonged course of broad-spectrum antibiotics can be a viable option for initial treatment of PG as long as there are no serious complications [19]. However, in cases which are unresponsive to medical treatment, surgery has been associated with reduced mortality rates (20% vs. 50% in patients undergoing medical treatment) and should be considered [13]. The combination of surgery and antibiotic therapy do not necessarily produce significantly different outcomes. In this case, given the rapid clinical deterioration and unresponsiveness to conservative measures, surgical resection became necessary with good patient outcomes [20]. Furthermore, rapid development of ACS, requires surgical decompression [16]. To our knowledge, this is only the sixth reported case of PG following routine endoscopic surveillance and random simple biopsy as part of a workup for anemia, and the only case reported with acute ACS as a complication [4–7].

Although, upper GI endoscopy is considered a very safe surgical procedure with minimal complication rates, complications still do occur. It is important to maintain a high degree of clinical suspicion in predisposed patients with post-procedural upper GI symptoms to ensure early diagnosis and prompt treatment. Most patients can be managed successfully with broad-spectrum antibiotics in the earlier stages. Complications, such as ACS, should be suspected in patients unresponsive to treatment, particularly in the presence of risk factors. Surgery is generally reserved for such cases to prevent more severe complications and death. In this case, a combination of surgery and antibiotics provided a favourable outcome.

## Data Availability

Not applicable.

## Abbreviations

PG: Phlegmonous gastritis
GI: Gastrointestinal
ACS: Abdominal compartment syndrome
WBC: White blood cell
CT: Contrast tomography
ICU: Intensive care unit
VAC: Vacuum assisted closure
IAH: Intra-abdominal hypertension
IAP: Intra-abdominal pressure
POD: Post-operative day
